# Flipside of the Coin: Iron Deficiency and Colorectal Cancer

**DOI:** 10.3389/fimmu.2021.635899

**Published:** 2021-03-11

**Authors:** Aysegül Aksan, Karima Farrag, Sami Aksan, Oliver Schroeder, Jürgen Stein

**Affiliations:** ^1^Institute of Nutritional Science, Justus-Liebig University, Giessen, Germany; ^2^Institute of Pharmaceutical Chemistry, Goethe University, Frankfurt, Germany; ^3^Interdisziplinäres Crohn Colitis Centrum, Rhein-Main, Frankfurt, Germany; ^4^DGD Kliniken Sachsenhausen, Frankfurt, Germany

**Keywords:** iron deficiency, iron deficiency anemia, colorectal cancer, immune host defense, intravenous iron therapy

## Abstract

Iron deficiency, with or without anemia, is the most frequent hematological manifestation in individuals with cancer, and is especially common in patients with colorectal cancer. Iron is a vital micronutrient that plays an essential role in many biological functions, in the context of which it has been found to be intimately linked to cancer biology. To date, however, whereas a large number of studies have comprehensively investigated and reviewed the effects of excess iron on cancer initiation and progression, potential interrelations of iron deficiency with cancer have been largely neglected and are not well-defined. Emerging evidence indicates that reduced iron intake and low systemic iron levels are associated with the pathogenesis of colorectal cancer, suggesting that optimal iron intake must be carefully balanced to avoid both iron deficiency and iron excess. Since iron is vital in the maintenance of immunological functions, insufficient iron availability may enhance oncogenicity by impairing immunosurveillance for neoplastic changes and potentially altering the tumor immune microenvironment. Data from clinical studies support these concepts, showing that iron deficiency is associated with inferior outcomes and reduced response to therapy in patients with colorectal cancer. Here, we elucidate cancer-related effects of iron deficiency, examine preclinical and clinical evidence of its role in tumorigenesis, cancer progression and treatment response. and highlight the importance of adequate iron supplementation to limit these outcomes.

## Introduction

Colorectal cancer (CRC) is the third most deadly and fourth most diagnosed cancer worldwide, and its incidence is steadily rising in developing nations (1). Both genetic characteristics and environmental factors play a role in intestinal carcinogenesis (2, 3). Alongside other well-established contributors, iron has recently emerged as a possible culprit in colorectal carcinogenicity (4). Published data support the hypothesis that excess oral iron intake is associated with an increased risk of CRC (5–8).

Iron is a vital micronutrient that has an essential role in many biological functions, in the context of which it has been found to be intimately linked to cancer biology (4, 9, 10). The trace element is required for energy production and intermediary metabolic actions as a catalyzer for REDOX-mediating enzymes. Proteins may bind directly to iron or contain iron in the form of heme or iron–sulfur clusters (11). Iron generates oxygen free radicals, which may in turn cause iron-induced apoptosis or ferroptosis. Furthermore, these iron-oxygen complexes are complicit in promoting mutagenicity and malignant transformation. Having undergone transformation, malignant cells require large quantities of iron in order to proliferate. Iron is also an important mediator of immune functions, including tumor surveillance carried out by the immune cells (9). Cytokine production in macrophages, a key aspect of host defense, is regulated by their iron content (11). Ideal iron intake must therefore be carefully balanced between iron deficiency and iron excess, since both can have potentially crucial clinical consequences with regard to cancer development. To date, however, although a large number of studies have comprehensively investigated and reviewed the role of excess iron in cancer initiation and progression (5, 9, 10, 12–14), potentially tumorigenic effects of iron deficiency have been largely neglected and are not yet well defined (4). This certainly deserves more research, since iron deficiency occurs particularly frequently in patients with CRC, both at the time of diagnosis and throughout the duration of disease (15–17).

Just as the effects of excess iron intake can potentially influence both the etiology and prognosis of CRC, so too can the physiological effects of iron deficiency (18–20). The risk of CRC has been found to be significantly elevated among patients with iron deficiency anemia (IDA) (15, 16, 21). Moreover, iron deficiency is evidentially associated with shorter survival times in patients with cancer (19). These findings are not surprising, since iron deficiency can limit hematopoiesis, a prerequisite for immune cell production, and iron is necessary for the correct functioning of the immune cells (22, 23). Thus, in cancer patients, iron deficiency can result in a diminished immune response and, consequentially, an impaired treatment response, a poor prognosis and reduced overall survival (18–20). In this review, we investigate the flipside of the coin regarding the role of iron in cancer, addressing consequences of iron deficiency on immune functions key to tumor development and progression, particularly in CRC, and elucidating current options for iron therapy to limit these outcomes.

## Definition of Iron Deficiency

Iron deficiency, with or without anemia, is the most frequent hematological manifestation in individuals with cancer, occurring in over 40% of patients. In patients with CRC, the reported rate is even higher, at around 60% (17, 24, 25). Two forms of iron deficiency can be observed in patients with cancer: absolute iron deficiency (AID) and functional iron deficiency (FID).

Whereas AID is characterized by depleted iron stores and inadequate iron supply, in FID, iron stores are adequate, but there is insufficient iron supply for erythropoiesis and other iron-dependent pathways (26, 27). The main cause of FID in cancer is the release of cancer-associated pro-inflammatory cytokines such as interleukin (IL)-6, IL-1, TNF-α, and IFN-γ. These cytokines upregulate hepcidin synthesis, thus reducing the quantity of iron released into the circulation (27–29). FID may also develop due to chemo- and/or radiotherapy-induced myelosuppression or increased erythropoiesis under therapy with erythropoiesis-stimulating agents (ESAs) (27, 29). Chronic kidney disease, a frequent comorbidity in cancer patients, can cause FID by reducing erythropoiesis and increasing levels of hepcidin (30, 31). FID is one of the major contributors to anemia of chronic disease (ACD), in this context also known as anemia of cancer or cancer-related anemia (29, 32).

In AID, on the other hand, iron stores are genuinely depleted. Nutritional deficiencies (e.g., malabsorption, tumor-induced anorexia, malnutrition) and especially manifest or occult blood loss, which are not uncommon in CRC, contribute to AID (26, 27, 29).

**Figure 1:**
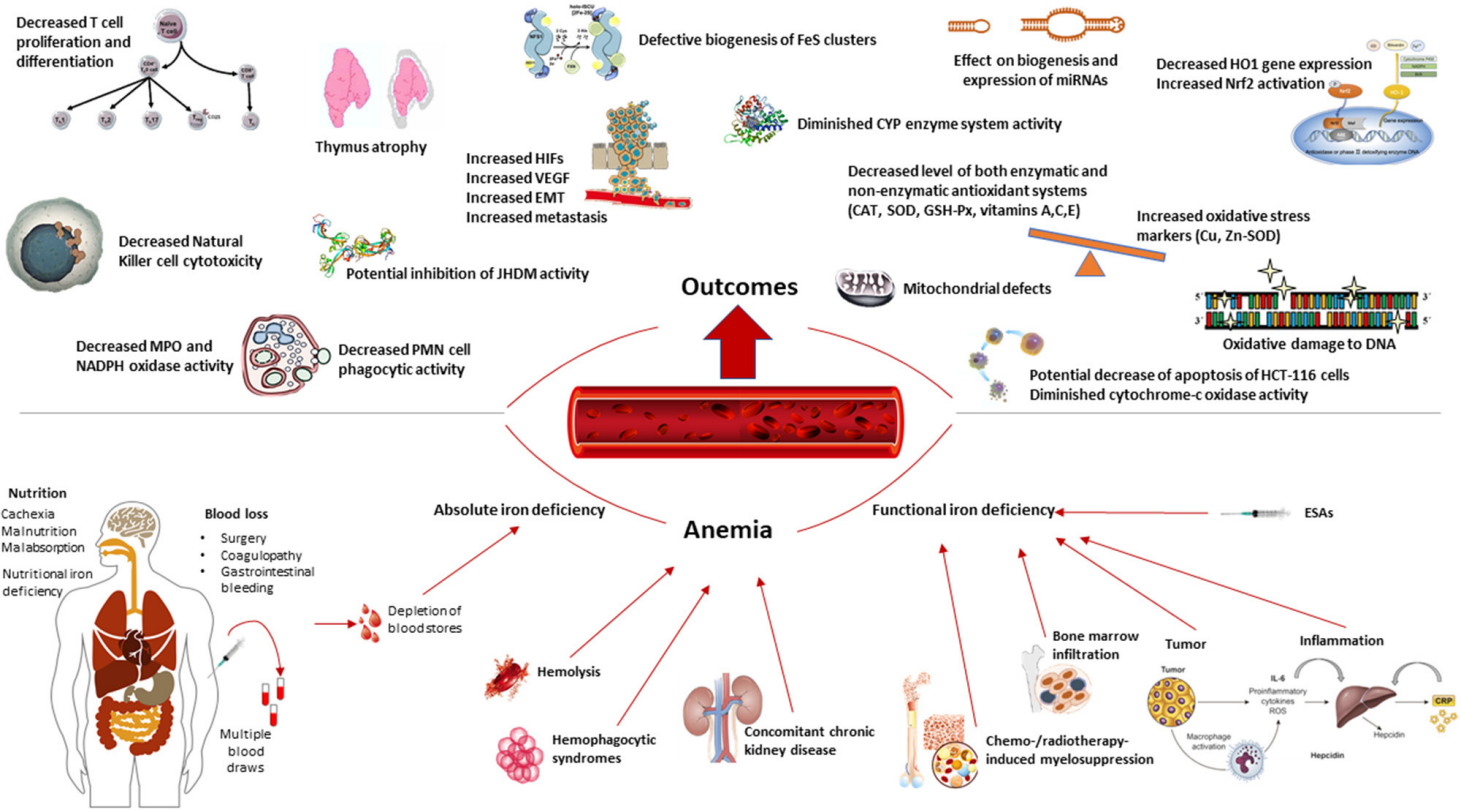
Overview of the consequences of iron deficiency and anemia in patients with cancer.

Figure 1 presents an overview of the consequences of iron deficiency and anemia in patients with cancer.

### Clinical Insight: Diagnosing Iron Deficiency in Patients With Cancer

Differentiation between AID and FID is essential, since the specific etiology of iron deficiency in patients with cancer is an important determinant of the treatment approach (26, 27, 33, 34).

Analysis of iron supply in the bone marrow with Perls' Prussian blue staining is the “gold standard” for diagnosis of iron deficiency (35). However, this technique is costly, highly invasive and non-automated, as a result of which it is largely impracticable in routine practice.

In healthy individuals, serum ferritin (SF) is recognized as a marker of iron stores, while other parameters, such as transferrin saturation (TSAT), mean corpuscular volume (MCV), percentage of hypochromic erythrocytes (%HYPO), Hb content of reticulocytes (CHr), soluble transferrin (sTfR), red blood cells (RBCs) and zinc protoporphyrin (ZnPP) reflect the amount of biologically available iron (26, 27). However, most of these parameters are altered in patients with cancer. Therefore, the differentiation of types of iron deficiency in this setting poses multiple challenges (27).

Iron deficiency is defined as transferrin saturation (TSAT) <20%, and can be further characterized as AID (SF < 100 ng/mL) or FID (SF > 100 ng/mL) (27, 33, 34). Since SF behaves as an acute-phase protein, due to the presence of cancer-related inflammation, its cut-off point is higher in patients with cancer than in persons without inflammatory disease (cut-off for SF in the latter is 30 ng/mL) (34). In addition to the more established markers TSAT and SF, ZnPP could represent a valuable addition to differential diagnostics, since it has been found to be increased in AID (34, 36, 37). While levels of soluble transferrin receptor (sTfR) have also been reported to be increased in AID and reduced in FID (27, 32), its levels may decrease following chemotherapy and increase after ESA treatment. Therefore, sTfR and markers related to sTfR, such as sTfR/log ferritin index, are less suitable as markers in an oncological setting (32, 34, 37). Other markers of iron deficiency, including CHr, %HYPO, MCV, and RBCs, fail to discriminate between AID and FID (34).

Measurement of circulating hepcidin could offer additional utility, not only in assessing iron status, but also in predicting response to iron therapy (38, 39). As yet, however, there is neither a validated clinical cut-off for hepcidin nor a simple standard test that would allow it to be fully used in clinical practice (27, 38).

## Impacts of Iron Deficiency on Cancer

Iron has anti-inflammatory and antioxidant properties and is vitally involved in functions of the immune system (4, 20, 40). It also plays an indispensable role in many other essential physiological processes, such as cell proliferation and differentiation, the maintenance of intestinal health, DNA synthesis and repair, and the metabolic breakdown of drugs and toxins (41–43). Iron homeostasis (23, 44, 45) and the role of iron in the initiation, progression and therapy of cancer have already been comprehensively reviewed in numerous publications (9, 10, 12, 13, 46). In this section, we specifically focus on the impacts of iron deficiency on CRC, from basic science to clinical outcomes (Figure 1).

### Iron Deficiency and Cancer Epigenetics

Epigenetic mechanisms have emerged as major actors that play diverse and important roles in the initiation and progression of cancer (47–49). While the role of iron in epigenetics has been described, the underlying mechanisms have not yet been thoroughly elucidated. Iron is essential for iron–sulfur (Fe-S) cluster synthesis in every cell of the body (50) and it is known that the key enzymes of DNA duplication, repair, and epigenetics have Fe-S clusters as prosthetic groups (50–54). Iron deficiency causes defective biogenesis of the Fe-S clusters, inducing DNA replication stress and genome instability, both of which are indications of malignant transformation (20, 54).

Jumonji-C (JmjC)-domain-containing histone demethylases (JHDMs) affect gene expression by demethylating lysine residues of histone tails, the most common sites of post-translational changes. Genetic alterations in JHDMs have been reported in various human cancers (55–57). Consequently, JHDMs are believed to be involved in oncogenesis (55). JHDMs are iron-dependent enzymes, having iron as a cofactor (51, 57). Therefore, iron deficiency might inhibit the activity of JHDMs, with possible oncologically relevant effects. Furthermore, hypoxia, a common feature of iron deficiency, has also been found to result in a loss of JHDM activity and probably contribute to changes in chemokine expression (56). The role of JHDMs can be two-sided, depending on the cancer type. Overall, therefore, it is important to maintain optimal iron levels (55).

The role of microRNAs (miRNAs), members of the noncoding RNA family, in the initiation, progression, metastasis and invasive activity of tumors has been characterized over the past decade. miRNAs are evolutionarily conserved, endogenous, single-stranded small RNAs of 18–22 nucleotides in length, that are encoded by eukaryotic genomic DNA. Aberrant expression of miRNAs may modify the normal expression of various genes including oncogenes and tumor-suppressor genes (47, 58). Ultimately, dysregulation of miRNA expression and related biological processes leads to poor outcomes in terms of cancer progression and development, and also to poorer therapeutic response (58–60). In addition, ~50% of miRNAs are located at genomic cancer-associated regions of loss of heterozygosity or loss of amplification and at fragile sites within chromosomes, underlining the important role of miRNAs in tumorigenesis (61).

Iron deficiency is suspected to affect miRNA biogenesis and expression and alter miRNA-mediated gene regulation networks by causing defective heme biosynthesis and degradation, hypoxia and increased ROS (62–66). Thus, iron deficiency can also increase the risk of tumorigenesis and lead to poor cancer prognosis and poor therapeutic outcomes by negatively influencing the gene regulation system of miRNAs (67).

Hypoxia, a common feature of iron deficiency, has been demonstrated to play a major part in tumor progression and treatment resistance in mice by corrupting the von Hippel-Lindau (VHL) gene, the master regulator of hypoxia-inducible factor (HIF) and thus a tumor suppressor (68). In iron-deficient, immunodeficient mouse xenograft models, the Notch signaling pathway was shown to be disrupted and expression of the transcription factor Snail elevated (69). Snail has numerous effects relevant to tumor growth, metastasis and treatment resistance: Its increased expression promotes cell motility and invasiveness by altering epithelial-mesenchymal transition (by repressing epithelial and enhancing mesenchymal markers). Furthermore, Snail endows stem cell-like characteristics on tumor cells, thus increasing therapy resistance (70).

Iron deficiency, through hypoxia, has been associated with enhanced expression of BCL2L1, the protein-coding gene that inhibits mitochondria-mediated cell death. Furthermore, iron deficiency has been shown to inhibit expression not only of CTSZ, the gene for the cysteine protease cathepsin Z, which has been associated with malignancy and inflammation, but also of CASP5, the gene for the cysteine peptide Caspase 5, which is involved in cellular apoptosis (71).

Iron deficiency is therefore associated with a variety of epigenetic changes and epigenetic mechanisms that are likely associated with oncogenesis. However, their role in cancer development and progression remains to be fully elucidated.

### Iron Deficiency and Pro-oxidant and Antioxidant Activities

It has been suggested that iron deficiency might cause an imbalance of the pro- and anti-oxidant systems (REDOX) (20). When iron is lacking, the level of both enzymatic and non-enzymatic antioxidant systems, such as catalase (CAT), superoxide dismutase (SOD), glutathione peroxidase (GSH-Px) and vitamins A, C, and E have been found to be decreased (6, 72–74). On the other hand, oxidative stress markers like Cu and Zn-SOD are increased (20, 75). These changes lead to an increased generation of reactive oxygen species (ROS), accompanied by a decrease in the body's total antioxidant capacity (74, 76–78). While ROS display varying reactivities toward different targets, they share the ability to damage cells by oxidizing proteins, lipids and DNA. This potential of ROS to cause cell damage and DNA mutation suggests that it may be directly or indirectly associated with tumor cell development, metastasis, tumor aggressiveness and treatment resistance as a reflection of accumulated ROS damage over time (20, 79, 80).

It has been demonstrated that by increasing oxidative stress, iron deficiency can cause damage to the mitochondria, corrupting mitochondrial DNA (81). Mitochondria are organelles of the cell that are primarily responsible for oxidative phosphorylation, the production of intracellular energy from oxygen and nutrients, as well as heme synthesis (82) and assembly of eukaryotic iron-sulfur (Fe-S) protein clusters (83). Mitochondria are also responsible for autoreproduction. Disruption of mitochondrial functions can therefore impair the integrity of the nuclear genome (84).

Hemoproteins are conjugated proteins with a variety of structures and functions that contain a non-protein component or prosthetic group called heme (or a derivative thereof). Increased ROS due to oxidative stress may induce the hemoproteins to discharge these heme groups, resulting in circulating free heme that can trigger additional production of free radicals. There are a number of mechanisms that can counteract pro-oxidant effects of free heme, such as rapid induction of heme oxygenase-1 gene (HMOX1) transcription and heme oxygenase-1 (HO-1) isoenzyme protein expression, which generates rapid catabolism of free heme in order to limit resultant cell damage (85, 86). As well as being involved in cellular homeostasis, HO-1 plays an important part in preventing oxidative tissue damage and mediating intracellular inflammatory mechanisms, apoptosis and cell proliferation (85). Lai et al. (87) reported that without adequate iron, HCT-116 human colon adenocarcinoma cells were unable to express the HO-1 gene completely, in response to toxicity. Since iron is essential for HO-1 gene expression, iron deficiency might lead to decreased cytoprotection through HO-1 expression (20).

Heme is an integral part of the CYP (intestinal cytochrome P450) antioxidant enzyme system (88–90). Iron deficiency has been shown to diminish CYP system activity in intestinal cells. Both in a xenograft murine model and in CRC cells, CYP2S1 gene depletion was identified to promote colorectal carcinogenesis (91–93). Thus, the effects of iron deficiency on heme synthesis can interfere with the CYP system, posing a risk factor for CRC.

*In vitro* studies in human brain cells have shown iron deficiency to result in significant reduction of the heme-containing electron transport protein (cytochrome-c oxidase/complex IV) (94). This has been shown to cause impairment of the heme metabolism, an increase in oxidative stress, and mitochondrial dysfunction (94). All of these are characteristic indications of cancer (20, 95).

The transcription factor Nrf2 (nuclear factor-E2-related factor-2) functions as a cellular sensor for oxidative stress. The genetic transcription of phase-II proteins via Nrf2 activation probably represents the most important signaling pathway for the body's immune response to oxidative stress and toxins. Nrf2 thus plays an essential role in cell protection. Iron deficiency has been found to activate autophagy and Nrf2 signaling for oxidative stress (96). Nrf2 activation has been implicated in cancer and is associated with a poor outcome and reduced survival in tumor types such as non-small cell lung cancer (97, 98). It has been proposed that constitutive activation of Nrf2 may encourage oncogenesis (99, 100) through actions promoting angiogenesis, metabolic reprogramming, chronic proliferation, and resistance to cell death (101, 102). Therefore, iron deficiency may promote oncogenesis by activating autography and Nrf2 signaling for oxidative stress.

### Iron Deficiency, Immune Response, and Cell Function

The interplay of iron homeostasis with cellular immune responses is complex and context dependent. Impairment of cellular immunity and antimicrobial activities of immune cells due to iron deficiency may create a microenvironment unconducive to the immunosurveillance mechanisms of the immune system that should identify and eliminate potential for malignant transformation. Furthermore, within the modified tumor microenvironment, immune cells may themselves exert a pro-tumorigenic response (4, 14, 20, 85).

The nuclear factor (NF-κB) and hypoxia-inducible factors (HIFs) are transcription factors that are critical to immune system regulation (103). The physiology of tumor cells allows them to grow and multiply rapidly and avoid apoptosis. Also characteristic of these cells are their capacities to ignore growth-inhibitory signals, to instigate angiogenesis, tissue invasion and metastasis, and to replicate infinitely. Almost all of the genes involved in the mediation of these processes are regulated by NF-κB transcription (104). Low levels of intracellular iron evidentially reduce phosphorylation of Re1A, a subunit of the NF-κB family of genes, and impair prolyl hydroxylation of HIFs (71, 105). Iron deficiency *per se* and iron deficiency-induced hypoxia can trigger the activation of HIFs, which are known to mediate cancer progression by upregulating target genes associated with angiogenesis and the metabolic reprogramming of tumor cells (106, 107), thus causing resistance to chemo- and radiotherapies (108, 109). HIF-1α plays a key role in the growth, progression and metastasis of solid tumors (110, 111). Iron deficiency has been found to promote HIF-1 transcription and inhibit HIF-2 transcription, thus corrupting the synergistic signaling pathways between the HIFs and NF-κB (71). Consequently, iron deficiency may weaken the immune response, increasing both the risk of oncogenesis and the probability of a poor prognosis and resistance to therapy when malignancy occurs.

Cellular iron depletion induced by the iron chelator desferoxamine mesylate (DFO) has been shown to increase HIF-1α (112). The transcription factor HIF-1α mediates expression of vascular endothelial growth factor (VEGF), a potent inducer of malignant angiogenesis and metastasis. Thus, iron deficiency has been reported to have important effects on HIF-1α stabilization, VEGF formation, angiogenesis and tumor progression in breast cancer, in both *in vitro* and *in vivo* studies (68, 113). Jacobsen et al. (114) found increased VEGF levels to be associated with a poor outcome in human renal cell carcinoma. Moreover, in one of these models, iron supplementation was found to significantly decrease VEGF levels in hypoxia, indicating a role for iron in counteracting HIF-1α stabilization and thus, possibly, in preventing angiogenesis (113).

Myeloperoxidase (MPO) and NADPH oxidase are enzymes that play a key role in interferon-γ (IFN-γ) induction by monocytes, and in microbial killing and phagocytosis by means of ROS production in neutrophils. These enzymes are iron dependent (115–118): Their catalytic activity is suppressed when iron deficiency is present, causing phagocytosis to be impaired. As a result, susceptibility to infections and tumor development may be increased (20, 118).

Natural killer (NK) cells are cytotoxic effector lymphocytes that perform unique functions including immunosurveillance and anti-tumor actions within the innate immune system (119). Hypoxia, which is characteristic of the iron deficient state, has been shown to inhibit the expression of vital activating NK-cell receptors and NK-cell ligands on tumor cell membranes (120, 121). Iron deficiency therefore disrupts the cytotoxic and specifically anti-tumor activities of NK cells and is conducive to oncogenesis and tumor growth.

Lymphocytes, comprising natural killer cells, T cells and B cells, are the major cellular constituents of cell mediated immunity. Cytotoxic T cells have several functions, one of which is the lysis of tumor cells. Iron deficiency has been shown to inhibit T cell proliferation and secretion of the potent anti-tumor cytokine IFN-γ (122). In murine models, iron deficiency was found to lead to atrophy of the thymus gland and the reduced excretion of CD28 thymocytes and spleen cells, causing impairment to lymphocytic motility and functions (123, 124). In addition, protein kinase-C translocation from cytosol to the plasma membrane, vitally necessary for T cell migration and immunological synapse, is reduced in the iron deficient state (125, 126). Furthermore, iron deficiency inhibits overall the expression of various diversely acting cytokines from cells of the immune system (127–129). Cell mediated immunity is therefore impaired due to iron deficiency, paving the way for cancer development and growth.

It has been demonstrated that intracellular iron plays a key role in apoptosis of HCT-116 (human cancer) cells (130). Furthermore, cytochrome-c oxidase activity, a significant marker of apoptosis resistance, is evidentially diminished in the presence of iron deficiency (131, 132). Therefore, the cancer-related effects of iron deficiency may influence not only tumor development and progression, but also apoptosis and treatment response.

## Evidence From Human Clinical Studies of Iron Deficiency Anemia in Relation to Colorectal Cancer

The abundant biological and immunological evidence describing important cancer-related effects of iron deficiency has direct implications for human health. Clinical and epidemiological studies have focused on various aspects of the relationship between iron deficiency and CRC, from etiology to progression and metastasis, therapeutic response and long-term outcomes.

Studies of patients with CRC found a significant association with low transferrin saturation in a cohort of Californian males (133) and with low serum ferritin in a case-control nested study of New York females (134). In another cohort study, men and postmenopausal women with iron deficiency without anemia had a five-fold and those with IDA a 31-fold increased risk of developing gastrointestinal cancer in comparison to individuals with normal hemoglobin (Hb) and TSAT levels (15).

In a large cohort of 965 men and women aged 50–75 years, Bird et al. (135) found a U-shaped relation between iron intake and colorectal polyps, with those consuming high (>27.3 mg/day) or low (<11.6 mg/day) quantities of iron more likely to develop colorectal polyps, a precursor lesion to CRC. In line with this, Cross et al. (136) showed that CRC risk was inversely associated with serum ferritin levels and positively associated with serum unsaturated iron binding capacity (UIBC). Moreover, serum iron and TSAT were found to have an inverse association with the risk of colon cancer, specifically (136). In a recent study by Hamarneh et al. (137) assessing risk factors for CRC following a positive fecal immunochemical test, IDA was reported as a significant risk factor for CRC [OR 7.93, 95% Cl (2.90–21.69), *p* < 0.001] independent of age. While the above findings suggest that iron deficiency could contribute to the pathogenesis of CRC, just as excessive iron intake does, the mechanisms are not yet fully understood. However, as presented above, preclinical research points to a role of iron deficiency in blunting the immune response, allowing tumor cell invasion under diminished immunosurveillance or switching to a pro-tumorigenic immune cell function in the tumor microenvironment (4, 9, 22, 23).

Not only may iron deficiency substantially influence oncogenesis, but it has also been found to influence oncological outcomes in patients with CRC. Zhen et al. (138) investigated long term effects of iron deficiency on the outcomes of 644 patients (19–83 years) with TNM stage II CRC and found IDA to be an independent predictor of long-term outcome in patients with T3N0M0 stage colon cancer. Patients with IDA had inferior outcomes and presented with worse tumor staging and lower disease-free survival than non-anemic patients (138). These findings suggest that IDA can influence CRC prognosis and outcomes, presumably by inhibiting immune system mechanisms that limit tumor growth, hindering responsiveness to treatments such as chemotherapy or surgery, and restricting the immune system's response to circulating tumor cells that can develop into distant metastasis (4, 9, 139). Lorenzi et al. (140) found that patients with both high and low serum ferritin levels who underwent curative or palliative surgery had shorter survival after a follow up period of at least 5 years in comparison to those with normal levels. Another study by An et al. (141) showed that patients with preoperative anemia treated with combined FOLFOX-based adjuvant chemotherapy had a worse prognosis than those without anemia. Additionally, a systematic review of 60 studies identified a 65% overall elevated mortality risk among cancer patients with anemia in comparison with those without anemia (19).

Overall, therefore, the evidence from epidemiological and clinical research corroborates data from preclinical studies, suggesting that iron deficiency, like iron surplus, might have a considerable negative influence with regard to oncogenesis, tumor progression and individual outcomes. Iron deficiency, with or without anemia, is associated with a poor prognosis, worse tumor staging, lower disease-free survival rates and a poorer response to oncological therapies in patients with CRC.

## On a Therapeutic Knife-Edge: Iron Replacement Therapy in Patients With Colorectal Cancer and Iron Deficiency/Anemia

There are currently three main treatment approaches for iron deficiency in the context of CRC; blood transfusions (RBC transfusions), erythropoiesis-stimulating agents (ESAs) and iron supplementation (26, 34). Since both RBC transfusions and ESAs are, like iron deficiency/anemia, independently associated with an increased risk of CRC recurrence and mortality (142–144), the use of iron substitution therapy to reverse anemia has gained more attention. In principle, iron can be replaced either orally or intravenously.

### Oral Iron

Oral substitution of iron has long been favored due to its simplicity and low costs, and as a result of lingering safety concerns due to adverse events associated with early intravenous iron compounds. However, its suitability in cancer patients is generally limited by concurrent inflammation, gastrointestinal discomfort and polypharmacy. Furthermore, oral iron has not been associated with consistent clinical or hematological improvement in patients with cancer (82, 145–147). On the contrary; it has been found to be ineffective in individuals with cancer and especially CRC, since intestinal iron absorption is greatly reduced in these patients (nearly 95% of the iron being excreted) (33). Furthermore, the increased availability of iron in the gut due to reduced intestinal iron absorption may support the proliferation of pathogenic gut bacteria conducive to tumor progression in preference to protective passenger bacteria that are more likely to hinder disease progression (148). As for the very small quantity of iron absorbed, most remains trapped within the enterocytes, where it is largely blocked by inflammatory cytokines and thus cannot be metabolized (33, 149). Overall, therefore, oral iron is unsuitable for iron replacement in patients with CRC.

### Intravenous Iron

Intravenous (IV) iron can overcome the absorptive inflammatory blockade of iron, since iron is directly captured by the macrophages (33). There is growing evidence to support benefits of IV iron therapy (without additional ESAs) in patients with cancer (150–160) and IV iron has been shown to optimize preoperative hemoglobin levels specifically in patients with CRC (158–163). On the other hand, in the extended IVICA trial, a randomized study including 116 patients with anemia and colorectal cancer treated preoperatively with oral or IV iron, no significant difference was found for 5-year overall survival or disease-free survival (164). There are some concerns about the possible role of iron overload in cancer, including promotion of tumor growth, enhanced oxidative stress and poor disease progression (165–167). Wilson et al. (168) suggest that “iron therapy may worsen colorectal tumor prognosis by supporting colorectal tumor growth and increasing the metastatic potential.” However, there is no direct evidence from experimental studies to substantiate this hypothesis and the clinical applicability of such experimental data in patients with cancer is limited, since they are based on high iron doses, differing routes of injection and a variety of iron formulations that are not typically used in clinical settings (27, 169). Furthermore, iron overload is rare in patients with cancer (34).

In rodent models of CRC induced by inflammatory or carcinogenic agents, whereas elevated oral iron intake was shown to increase the incidence of tumors, systemic (IV) iron supplementation did not have the same effect (170, 171). This suggests that increased luminal iron, but not systemic iron levels, increase colorectal carcinogenesis in inflammatory models of CRC (172, 173). Radulescu et al., who showed in a rodent model that luminal iron cooperates with Apc (*adenomatous polyposis coli gene*) loss to promote intestinal tumorigenesis, propose that in patients with CRC, a combination of colonic luminal iron chelation and concurrent systemic iron replacement therapy would both resolve anemia and at the same time diminish the carcinogenic pool of residual iron within the colon (174).

Evidence from prospective clinical trials describing outcomes of IV iron therapy (alone or in combination with ESAs) in an oncological population are relatively scarce but their results are in line with the findings of rodent model studies. Short-term studies are reassuring, having not shown increased tumor progression in patients treated with IV iron and ESAs (34). One prospective randomized controlled trial evaluating treatment with IV iron and ESAs in patients with cancer (175), with a median follow-up period of 1.4 years, failed to find any negative effects on long-term outcomes or survival. A retrospective cohort study of patients who underwent surgery for CRC, with an extended follow-up period (median 3.9 years), confirmed that overall and disease-free survival did not significantly differ in subjects treated with IV iron (in this case, ferric carboxymaltose at a dose of 1,000–2,000 mg) as compared with a matched group not receiving IV iron (176). A comprehensive review of iron dextran use by Gilreath et al. concluded that there was no clinical evidence to support an elevated risk of cancer growth due to iron overload (167).

Regarding the risk of infections, no alarming signs have emerged in patients with cancer treated with IV iron. Nevertheless, given the role of iron in immune response and microbial proliferation (177), current guidelines prudently advise that IV iron should not be administered to patients who have, or are suspected to have, active infections (34).

No increase in cardiovascular morbidity has been observed in connection with IV iron therapy (82, 145, 178–180). However, it is recommended to avoid concomitant administration of IV iron and cardiotoxic chemotherapy: IV iron should be administered either before or after application of chemotherapy, or at the end of the chemotherapy treatment cycle (34).

## Conclusion

In contrast to the large amount of research already dedicated to the effects of excess iron as a probable (co-)trigger and driver of oncogenesis, the role of iron deficiency has been largely neglected and—on the evidence of the reviewed preclinical and clinical data—possibly underestimated. In particular, iron is vital for optimal functioning of the immune system, playing major roles in a multitude of different immune processes and pathways. Iron deficiency influences crucial mechanisms such as immune surveillance, gene regulation and cell apoptosis, all of which are key to host defense against malignant transformation and tumor growth. Clinical studies in patients with cancer and iron deficiency/anemia suggest that that unlike oral iron, IV iron therapy (with/without ESAs) improves overall outcomes without increasing risk of infection or cardiovascular morbidity. Excess (uningested/residual) oral iron can cause oncogenic effects in the intestinal tract and is thus generally unsuitable for patients with CRC (although its use may occasionally be justified, employing “defensive” dosing strategies). In general, IV iron does not appear to have this potential for local exacerbation, as confirmed by rodent studies. Iron overload is rarely seen in patients with cancer and there is no clinical evidence that IV iron negatively affects tumor progression. Nevertheless, in view of the abounding evidence of effects of iron overload on tumor growth, we suggest that IV iron should be cautiously supplemented with the goal of avoiding anemia and maintaining iron stores. Additional research is needed to confirm the appropriateness of IV iron replacement in patients with cancer, to explore the feasibility of concurrent luminal iron chelation, to determine target levels for iron store maintenance, and to shed further light on the effects of chronic iron deficiency on iron-dependent mechanisms in the context of the tumor microenvironment.

## Author Contributions

AA performed the initial literature search. All authors participated in the data analysis, preparation of the manuscript draft, reviewed the manuscript for important intellectual content and approved the final version for submission.

## Conflict of Interest

AA: Congress expenses, consultancy and lecturing fees: Vifor Pharma and Immundiagnostik AG. KF: Speakers' honoraria: Immundiagnostik AG. OS: Congress expenses: Abbvie, Falk, Janssen, Takeda; Lecturing fees: Abbvie, Falk, Janssen, Takeda, Norgine, Pfizer. JS: Consulting: Pharmacosmos, Vifor. Abbvie, Bristol Myers Squibb, Dr Schär, Falk, Ferring, Fresenius Kabi, Immundiagnostik, Janssen, Medice, MSD, Pfizer, Shire, Takeda, Thermofisher. Board member: Pharmacosmos, Vifor, Abbvie, Bristol Myers Squibb, Dr Schär, Ferring, Fresenius Kabi, Immundiagnostik, Janssen, MSD, NPS, Takeda, Shield. Lecturing: Vifor. The remaining authors declare that the research was conducted in the absence of any commercial or financial relationships that could be construed as a potential conflict of interest.

## Abbreviations

AID: absolute iron deficiency
CAT: catalase
CHr: hemoglobin content of reticulocytes
CRC: colorectal cancer
EMT: epithelial to mesenchymal transition
ESA: erythropoiesis-stimulating agent
Fe-S cluster: iron-sulfur cluster
FID: functional iron deficiency
GSH-Px: glutathione peroxidase
Hb: hemoglobin
HIF: hypoxia-inducible factor
IDA: iron deficiency anemia
IFN: interferon
IL: interleukin
JHDM: Jumonji-C (JmjC)-domain-containing histone demethylase
MCV: mean corpuscular volume
MiRNA: microRNA
NF: nuclear factor
NK: natural killer
MPO: myeloperoxidase
RBC: red blood cell
REDOX: oxidation-reduction
SF: serum ferritin
SOD: superoxide dismutase
TNF: tumor necrosis factor
TSAT: transferrin saturation
ZnPP: zinc protoporphyrin
sTfR: soluble transferrin receptor
UIBC: unsaturated iron binding capacity
VEGF: vascular endothelial growth factor
VHL: von Hippel-Lindau
%HYPO: percentage of hypochromic erythrocytes.
